# A distinct subset of human NK cells expressing HLA-DR expand in response to IL-2 and can aid immune responses to BCG

**DOI:** 10.1002/eji.201041180

**Published:** 2011-04-14

**Authors:** J Henry Evans, Amir Horowitz, Maryam Mehrabi, Emma L Wise, James E Pease, Eleanor M Riley, Daniel M Davis

**Affiliations:** 1 Division of Cell and Molecular Biology, Imperial College London, South Kensington CampusLondon, UK; 2 Department of Immunology and Infection, Faculty of Infectious and Tropical Diseases, London School of Hygiene and Tropical MedicineLondon, UK; 3 National Heart and Lung Institute, Imperial College London, South Kensington CampusLondon, UK

**Keywords:** Cellular crosstalk, HLA-DR, IL-2, Immune responses, NK cells

## Abstract

Subsets of NK cells can have distinct functions. Here, we report that >25% of human peripheral blood NK cells express HLA-DR after culture with IL-2. This can be driven by an expansion of a small subset of NK cells expressing HLA-DR, in contrast to previous assumptions that HLA-DR is upregulated on previously negative cells. HLA-DR-expressing NK cells showed enhanced degranulation to susceptible target cells and expressed chemokine receptor CXCR3, which facilitated their enrichment following exposure to CXCL11/I-TAC. Suggesting HLA-DR-expressing NK cells have an important role in an immune response, stimulation of PBMCs with *Mycobacterium bovis* BCG (BCG) triggered expansion of this subset. Importantly, the magnitude of an individual's NK cell IFN-γ response triggered by BCG was associated with the initial frequency of HLA-DR-expressing NK cells in PBMCs. More directly indicating the importance of HLA-DR-expressing NK cells, enriching the frequency of this subset in PBMCs substantially augmented the IFN-γ response to BCG. Thus, HLA-DR expression marks a distinct subset of NK cells, present at low frequency in circulating blood but readily expanded by IL-2, that can play an important role during immune responses to BCG.

## Introduction

NK cells are lymphocytes of the innate immune system that can directly kill transformed or virally infected target cells and are a key source of a variety of cytokines, including IFN-γ. However, NK cells are heterogeneous in phenotype with different subsets expressing different combinations of surface receptors 1–3. The best studied distinctions between NK cell subsets include CD56^dim^ and CD56^bright^ populations 4, 5, and the expression of different chemokine receptor profiles 6, 7. Differences in the expression profiles of chemokine receptors may suggest differential trafficking of NK subsets, which likely relate to their functional roles. It has been established that NK cells trafficking to lymph nodes can interact with DCs 8, for example.

Unlike the class I MHC protein, which is expressed on most human cells, expression of class II MHC proteins is usually considered as being restricted to professional APCs. However, expression of HLA-DR on NK cells has also been documented, where it has sometimes been used as an activation marker because of its increased expression following IL-2 stimulation 9–17. HLA-DR-expressing NK cells have been shown to present tetanus toxin and house dust mite-derived peptides to T-cell clones in an HLA-DR-restricted manner 18. Antigen bound by activating NK receptors can be internalised to class II MHC-loading compartments and processed for subsequent HLA-DR-mediated presentation leading to T-cell proliferation 19. In addition, HLA-DR-expressing NK cells have been found at elevated frequencies in inflammatory situations such as chronically inflamed tonsil tissue and decidual NKs from CMV-infected mothers 19. An early study also found a correlation between the percentage of NK cells expressing HLA-DR and the level of NK cell-derived IFN-γ transcript, suggesting a pro-inflammatory role for these NK cells 20. Thus, NK cells have the ability to present antigen, at least in certain circumstances, and here we set out to address how HLA-DR is expressed on activated populations of NK cells and to test the importance of the subset of NK cells that express HLA-DR in immune responses of whole PBMCs.

We found that a distinct subset of circulating HLA-DR-expressing NK cells in the peripheral blood preferentially proliferate in response to IL-2 leading to their enrichment. This is in contrast to the previous assumption that HLA-DR is expressed on activated cells that were initially HLA-DR negative. We found that HLA-DR-expressing NK cells exhibit greater lytic capacity and express a chemokine receptor associated with both recruitment to sites of inflammation and homing to the lymph node. In addition, we set out to test whether or not this subset of NK cells could be important in immune responses. We used *Mycobacterium bovis* BCG (BCG) as a model pathogen, noting its common use as a vaccine and, importantly, because of the heterogeneous NK cell response that it elicits 21. HLA-DR-expressing NK cells were expanded in the response of PBMCs to BCG. Artificially enriching the HLA-DR-expressing compartment of NK cells substantially enhanced the response to BCG, showing that HLA-DR-expressing NK cells can play a significant role during the initiation and amplification of inflammatory responses.

## Results

### A distinct subset of human peripheral blood NK cells expressing HLA-DR expand after IL-2 stimulation

**Figure 1:**
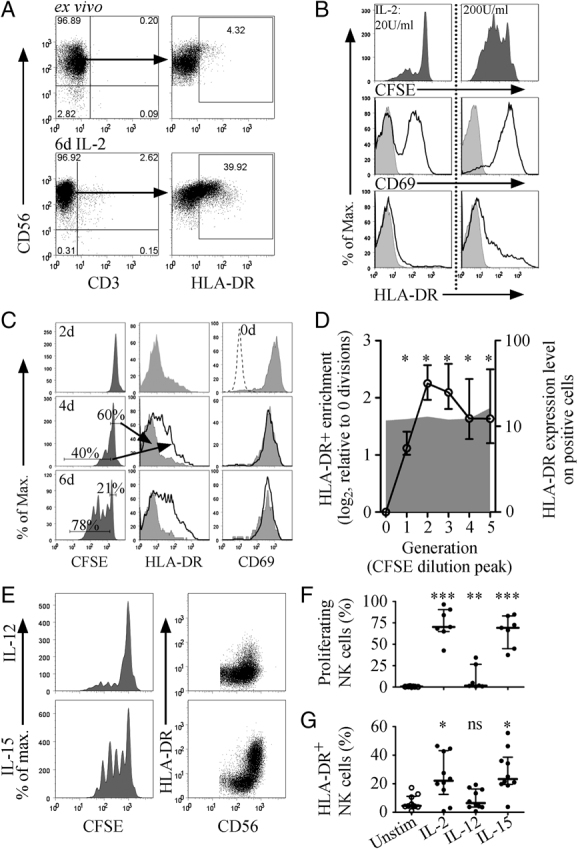
A small circulating population of NK cells expressing HLA–DR expands with IL-2. (A) Freshly isolated NK cells were analysed ex vivo (top), and after 6 days culture with IL-2 (bottom), by flow cytometry for expression of HLA-DR on CD56^+^ CD3^−^ NK cells. Plots for one donor (representative of >10 donors), showing CD56^+^ CD3^−^ gates (left) and HLA-DR expression on the NK cells (right), gates show percentage HLA-DR^+^. (B) Freshly isolated human NK cells were labelled with CFSE and cultured for 6 days with rIL-2 at 20 U/mL (left) or 200 U/mL (right), then analysed by flow cytometry. Plots show proliferation by CFSE (top), expression of CD69 (middle) and HLA-DR (bottom), representative of two donors. (C) Freshly isolated human NK cells were labelled with CFSE and cultured for up to 6 days with rIL-2 (200 U/mL). NK cells were analysed by flow cytometry for expression of HLA-DR (middle) and CD69 (right) on non-proliferating (grey shaded) and proliferating (black line) NK cells as gated in the CFSE histogram (left). The CD69 plot for 2 days stimulation also includes the expression level ex vivo (dashed line). (D) Data for six donors comparing the percentage of HLA-DR^+^ NK cells (left axis, circles and black line) and the level of expression (geomean) of HLA-DR (right axis, grey shaded area) between each generation (distinct CFSE peaks). The percentage of HLA-DR^+^ NK cells is shown as a fold increase over the unproliferated cells, median±interquartile ranges, ^*^p<0.05, tested by Wilcoxon matched pairs. The level of expression was calculated as the fold increase in the geometric mean of the HLA-DR^+^ NK cells relative to the total NK population. (E) Freshly isolated NK cells were labelled with CFSE and cultured with rIL-2, rIL-12 or rIL-15 for 6 days, then analysed for expression of HLA-DR by flow cytometry. Data from a representative donor showing CFSE profiles (left) and corresponding HLA-DR expression on CD56^+^ NK cells (right) for stimulation by IL-12 (top) and IL-15 (bottom). (F) Data for seven donors showing percentage of NK cells that had undergone at least one division and (G) the percentage of HLA-DR^+^ NK cells, ^*^p<0.05, ^**^p<0.01, ^***^p<0.001, tested by Wilcoxon matched pairs, median±interquartile range.

After 6 days culture in IL-2, 25–41% (median 34%) of CD3^−^CD56^+^ primary NK cells, derived from peripheral blood, expressed HLA-DR. In contrast, only 2–5.5% (median 3.5%) of fresh NK cells ex vivo expressed HLA-DR (Fig. 1A and Supporting Information Fig. S1A). This is consistent with previous observations 9, 22. The expression of HLA-DR on freshly isolated NK cells is unlikely to be a result of incidental activation in vivo because it is not co-expressed with the activation marker CD69 on NK cells in fresh PBMCs (Supporting Information Fig. S2A). By comparing the expression of HLA-DR and the activation marker CD69 on NK cells cultured for 6 days with a standard (200 U/mL) or sub-optimal (20 U/mL) dose of recombinant (r)IL-2, we found that the proportion of HLA-DR-expressing NK cells only increased at the higher dose, while CD69 was upregulated at both doses. Proliferation of NK cells, monitored by CFSE dilution, was far higher with 200 U/mL IL-2 (Fig. 1B and Supporting Information Fig. S1B and S2B) and therefore we next set out to test whether NK cell proliferation and HLA-DR expression were directly related.

The proportion of HLA-DR-expressing NK cells increased gradually over 6 days of culture and was confined to cells in which CFSE had been diluted, i.e. the proliferating cells (Fig. 1C and 1D, Supporting Information Fig. S1C). The level of expression of HLA-DR on NK cells remained constant and it was the proportion expressing HLA-DR that increased in proliferating cells (Fig. 1D). Interestingly, while both IL-12 and IL-15 activate NK cells, only IL-15 induced significant proliferation and increased HLA-DR expression (Fig. 1E and 1G, Supporting Information Fig. S1D and S1E). In contrast, CD69, an early activation marker on NK cells 23, was expressed on over 95% of the NK cells after only 48 h (Fig. 1C). Clearly, expression of HLA-DR is not simply a marker of NK cell activation, as has been suggested previously 9–17. These data suggest instead that the increase in proportion of NK cells expressing HLA-DR, in response to IL-2 stimulation, is due to preferential expansion of a small circulating population of HLA-DR^+^ cells, rather than de novo expression on previously non-expressing cells.

### Expression levels of HLA-DR are clonally restricted on NK cells

**Figure 2:**
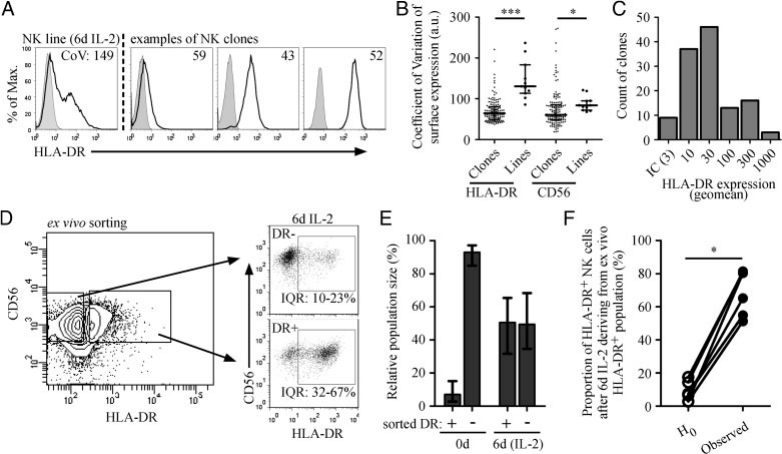
Expression levels of HLA-DR on NK cells are clonally restricted. NK cell clones were analysed by flow cytometry for expression of CD56 and HLA-DR. (A) The expression of HLA-DR on a polyclonal NK cell line after 6 days IL-2 culture (left) was compared with expression on individual clones derived from the same donor; three examples are shown (right). The coefficient of variation (CoV) of the expression of HLA-DR was calculated for each clone and cell line (indicated in top right of histograms). (B) Coefficient of variation data for expression of HLA-DR and CD56 from 143 clones from four independent donors, ^*^p<0.05, ^***^p<0.0005, tested by Mann Whitney, median±interquartile range. (C) The expression level (geomean) of HLA-DR was analysed on 129 clones from four donors. (D) Freshly isolated human NK cells were sorted into CD56^+^/HLA-DR^−^ (DR^−^) and CD56^+^/HLA-DR^+^ (DR^+^) populations. The sorted populations were cultured for 6 days with IL-2, then analysed by flow cytometry for expression of HLA-DR and CD56. Numbers beneath gates indicate interquartile ranges (IQR) of proportions of HLA-DR-expressing NK cells from six donors. (E) The relative population sizes of the sorted NK cell populations (−/+) were compared during sorting (0 days) and after 6 days culture with IL-2 (6d IL-2). Graphs show median±interquartile range. (F) The proportion of HLA-DR-expressing NK cells after 6 days culture with IL-2 that derived from those that initially expressed HLA-DR ex vivo was calculated. This was compared to a null hypothesis (H_0_) in which no preferential proliferation and no clonal restriction of HLA-DR expression was assumed. *n*=6, ^*^p<0.05 tested by Wilcoxon matched pairs.

We next tested this hypothesis using NK cell clones, i.e. where each population of cells was expanded from a single seed cell. In IL-2 cultured polyclonal NK cell populations, there was a broad range of HLA-DR expression, including some HLA-DR-negative NK cells (Fig. 1 and 2A). In contrast, HLA-DR expression was far more homogeneous within individual NK cell clones (examples shown in Fig. 2A). Specific comparison of the coefficients of variance of the HLA-DR expression showed that levels of HLA-DR expression were significantly less variable in NK cell clones than in NK cell lines (*p*=0.0004, Fig. 2A and B). Although the absolute expression level of HLA-DR varied between clones (Fig. 2A and C), only 6% of NK cell clones did not express HLA-DR (Fig. 2C).

### HLA-DR expression marks a subset of NK cells that exhibit enhanced proliferation

Supporting the proliferation studies (Fig. 1), the analyses of NK cell clones (Fig. 2A–C) suggest that HLA-DR-expressing NK cells proliferate more readily in response to IL-2. Thus, we explicitly compared the proliferative potential of HLA-DR^−^ and HLA-DR^+^ NK cells. Freshly isolated NK cells were sorted into CD56^+^/HLA-DR^−^ and CD56^+^/HLA-DR^+^ populations and cultured for 6 days with IL-2. After culture, the sorted NK cell populations were analysed by flow cytometry for expression of HLA-DR (Fig. 2D). The cultured populations derived from sorted HLA-DR^+^ NK cells maintained a significant three-fold greater proportion of NK cells expressing HLA-DR (*p*=0.03, Fig. 2D). More importantly, there was a marked difference in the rates of proliferation of these two sorted populations. Immediately after sorting, the HLA-DR^−^ population was 13-fold larger than the HLA-DR^+^ population. However, after 6 days culture with IL-2 the sorted populations were of equal size (Fig. 2E). Thus, the proliferation rate of the NK cells in peripheral blood that express HLA-DR is significantly greater than HLA-DR^−^ NK cells. This confirmed earlier results that the HLA-DR-expressing NK cells preferentially proliferate in response to IL-2 stimulation.

We used these data to calculate the percentage of HLA-DR-expressing NK cells in a polyclonal population after 6 days culture with IL-2 that derived from the small initial population of HLA-DR-expressing NK cells ex vivo. This was calculated by factoring the percentage of HLA-DR^+^ NK cells in each sorted population by the relative population size, and thus calculating what contribution each sorted population makes to the total of HLA-DR^+^ NK cells after 6 days of culture with IL-2. From our experimental observations, 54–81% (median 73%) of the HLA-DR-expressing NK cells after 6 days culture with IL-2 derived from those that express HLA-DR ex vivo. This is significantly higher than would be predicted from a model with no preferential proliferation or clonal restriction of HLA-DR expression (*p*<0.05, Fig. 2F). Thus, HLA-DR expression marks an NK cell phenotype that exhibits enhanced proliferation in response to IL-2 or IL-15 stimulation. This preferential expansion of an initially small circulating population of HLA-DR-expressing NK cells drives, in part, the increase in HLA-DR expression following IL-2 or IL-15 stimulation. As well as being of fundamental importance to NK cell biology this observation is important practically because it implies that studies of cultured NK cell clones, and IL-2 cultured NK cell lines, will be primarily focused on the phenotype of this specific HLA-DR-expressing subset of NK cells.

### HLA-DR-expressing NK cells exhibit enhanced degranulation

**Figure 3:**
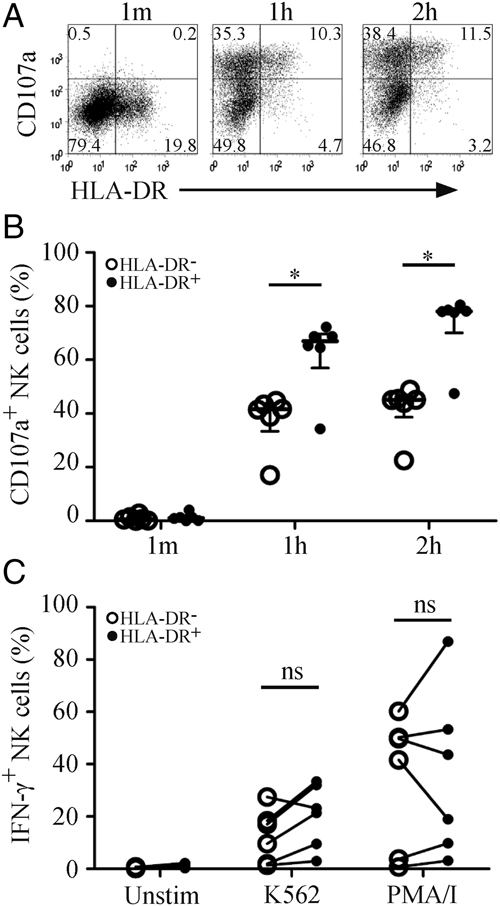
HLA-DR^+^ NK cells exhibit greater degranulation. (A) NK cells were cultured for 6 days with IL-2 and then tested for degranulation against K562 target cells over 1 min, 1 h or 2 h. NK cells were gated by HLA-DR expression, representative of six donors. (B) Degranulation (CD107a^+^) was compared between HLA-DR^−^ and HLA-DR^+^ NK cells for six donors, ^*^p<0.05, tested by Wilcoxon matched pairs, median±interquartile range. (C) NK cells from the same donors were incubated with K562 target cells or stimulated with PMA/ionomycin for 5 h, then analysed by flow cytometry for intracellular IFN-γ. Percentage of IFN-γ^+^ NK cells was compared between HLA-DR^−^ and HLA-DR^+^ NK cells, tested by Wilcoxon matched pairs.

To test how degranulation and cytokine secretion compared for HLA-DR positive and negative NK cells, staining for LAMP-1/CD107a and intracellular IFN-γ production was assessed for NK cells co-incubated with the target cell, K562 24. While only 45% of HLA-DR^−^ NK cells degranulated, over 75% of HLA-DR^+^ NK cells responded (*p*=0.03, Fig. 3A and B and Supporting Information Fig. S4A). HLA-DR-expressing NK cells had a slightly increased capacity for IFN-γ secretion upon stimulation by K562, 22% compared with 13% for HLA-DR^−^ NK cells, but this was not statistically significant (*p*=0.09, Fig. 3C and Supporting Information Fig. S4B). No such distinction was seen when the cells were activated with PMA/ionomycin (Fig. 3C), demonstrating that HLA-DR^+^ and HLA-DR^−^ NK cells have the same intrinsic capacity for IFN-γ production.

### HLA-DR-expressing NK cells are enriched by chemotaxis via the chemokine receptor CXCR3

**Figure 4:**
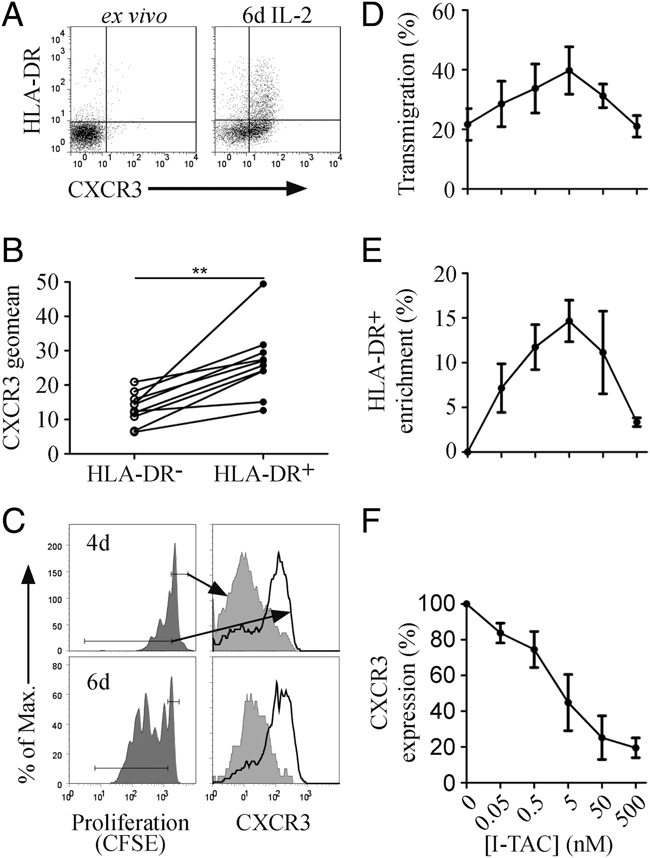
HLA-DR^+^ NK cells express a functional chemokine receptor CXCR3. (A) NK cells from one donor ex vivo (left) and after 6 days culture with IL-2 (right) were analysed by flow cytometry for expression of HLA-DR and CXCR3. (B) NK cells cultured with IL-2 for 6 days were analysed by flow cytometry for expression of HLA-DR and CXCR3. The intensity (geomean) of CXCR3 expression was compared between HLA-DR^−^ and HLA-DR^+^ populations. (C) Comparison of CXCR3 expression (right) on non-proliferating (grey shaded) and proliferating (black) NK cells as gated in the CFSE histogram (left). (D) NK cells from three donors were cultured with IL-2 for 6 days, then assessed for their chemotactic response to the CXCR3 ligand I-TAC in a transmigration chemotaxis assay. The percentage of NK cells that responded was calculated by haemocytometer — counting the cells in the lower chamber. (E) The transmigrated cells were also analysed by flow cytometry for expression of HLA-DR, plotted as the enrichment of HLA-DR^+^ NK cells in the transmigrating cells, relative to the buffer control, and (F) expression of CXCR3 on transmigrated NK cells. (D–F) *n*=3, median±range.

The chemokine receptor CXCR3 has previously been shown to be expressed on a subset of NK cells 6, 7, on some T cells 7 and on conventional APCs 25, 26, and facilitates their migration to secondary lymphoid organs 8, 27. This chemokine receptor is also important for lymphocyte recruitment to sites of inflammation 28. Here, co-staining NK cells for HLA-DR and CXCR3 demonstrated that the majority of NK cells that express HLA-DR following culture with IL-2 also express CXCR3, although this association was not observed on the HLA-DR-expressing NK cells ex vivo and so may reflect de novo expression (Fig. 4A and B). Expression of CXCR3 was higher on IL-2-activated NK cells in which CFSE was diluted (Fig. 4C), i.e. on proliferating cells, as seen for HLA-DR (Fig. 1C). Importantly, HLA-DR-expressing NK cells migrated in a dose-dependent manner to CXCL11/I-TAC, a cognate chemokine for CXCR3 (Fig. 4D). This transmigration led to a modest enrichment of the HLA-DR-expressing subset in cells responding optimally to CXCL11/I-TAC (Fig. 4E). This enrichment corresponded to the peak transmigration (Fig. 4D and E). Furthermore, activation of CXCR3 on NK cells could be detected via internalisation of the receptor upon recognition of CXCL11/I-TAC, in a dose-dependent manner (Fig. 4F). These data are consistent with HLA-DR-expressing NK cells being recruited to sites of inflammation 10, and being able to migrate to lymph nodes via CXCR3, which could explain the unexpectedly high proportion of HLA-DR-expressing NK cells found in human lymph nodes 22.

### HLA-DR-expressing NK cells expand in response to BCG stimulation

**Figure 5:**
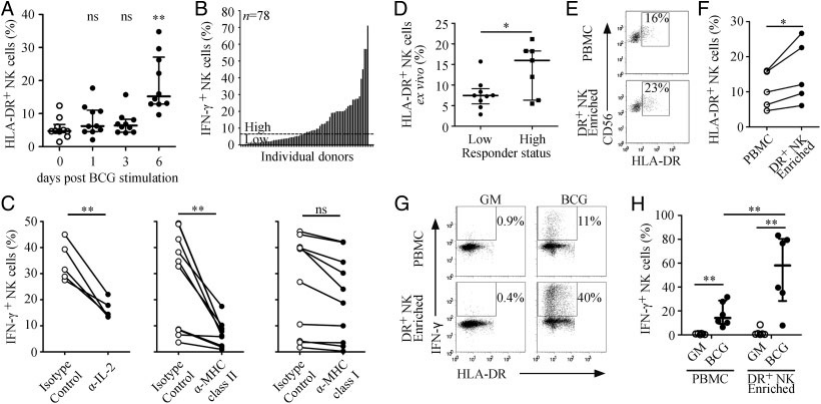
Enrichment of HLA-DR^+^ NK cells enhances the IFN-γ response to BCG. (A) PBMCs from ten donors were cultured with BCG for up to 6 days and the percentage of CD56^+^CD3^−^ NK cells staining positive for HLA-DR was measured by flow cytometry, median±interquartile range, ^**^p<0.01 tested by Wilcoxon matched pairs. (B) PBMCs from 78 donors were cultured with BCG for 24 h then analysed by flow cytometry for intracellular IFN-γ in NK cells, dotted line represents the median (6.5%). (C) PBMCs from ten donors were cultured with BCG for 24 h with blocking antibodies to MHC molecules or to IL-2 or with isotype matched controls. The percentage of IFN-γ^+^ NK cells was determined by flow cytometry, ^**^p<0.01 tested by Wilcoxon matched pairs. (D) PBMCs from 16 donors (seven high and nine low responders) were analysed by flow cytometry for the percentage of HLA-DR-expressing NK cells, median±interquartile range, ^*^p<0.05 tested by Mann Whitney one-tailed test. (E) PBMCs were enriched for HLA-DR^+^ NK cells and confirmed by flow cytometry; gates indicate percentage of HLA-DR^+^ NK cells. (F) HLA-DR-expressing NK cell enrichment for five donors, ^*^p<0.05 tested by Mann Whitney. (G) PBMCs (top) or HLA-DR^+^ NK cell-enriched PBMCs (bottom) were cultured for 24 h with growth medium control (GM, left) or with BCG (right) then analysed by flow cytometry for intracellular IFN-γ in CD56^+^CD3^−^ NK cells; gates indicate percentage of IFN-γ^+^ NK cells. (H) Data for five donors, median±interquartile range, ^**^p<0.01 tested by Wilcoxon matched pairs.

To explore the importance of these findings in the context of an infectious agent, we investigated the response of NK cells to BCG. BCG stimulation of whole human PBMCs for up to 6 days resulted in a three-fold increase in the proportion of NK cells expressing HLA-DR. Consistent with results obtained for IL-2 stimulation of isolated NK cells, this expansion was only significant after 6 days (*p*<0.01, Fig. 5A). Interestingly, previous studies have demonstrated that CD56^bright^ NK cells constitute the majority of proliferating lymphocytes after 6 days stimulation of PBMCs with extracellular BCG 29, 30. Following stimulation of PBMCs with BCG, the NK cells produce IFN-γ, similar to findings reported previously for PBMCs stimulated with *Plasmodium falciparum* infected erythrocytes 31 or with *Leishmania major* 32. Interestingly, the proportion of NK cells producing IFN-γ varies considerably between donors (Fig. 5B and Supporting Information Fig. S5). From 78 donors analysed, the median proportion of IFN-γ^+^ NK cells was 6.5%. Strikingly, secretion of IFN-γ by NK cells could be blocked by a neutralising mAb against IL-2 (Fig. 5C). Moreover, depleting CD3^+^ or CD4^+^ cells abrogated the response. Thus, T cells, shown to produce IL-2, were specifically required (Supporting Information Fig. S6). Interestingly, as shown previously for *P. falciparum* stimulation, the NK cell response was also significantly reduced by blocking class II but not class I MHC proteins (Fig. 5C), supporting the emerging concept of NK cells as regulators of an adaptive immune response 32–34. Together, these data are consistent with HLA-DR-expressing NK cells playing a role in establishing the immune response to BCG and we next set out to test this specifically.

### HLA-DR-expressing NK cells aid the immune response to BCG

Understanding the heterogeneity in human responses to pathogens is an important new frontier in contemporary immunology. The specific designation of low and high IFN-γ secretion responses to malaria has been noted previously 34–36. Here, we classified donors as “low” responders to BCG if fewer than 6.5% of their NK cells stained positively for IFN-γ and as “high” responders if more than 6.5% did so. We then analysed the frequency of HLA-DR-expressing NK cells in PBMCs from low (*n*=9) or high (*n*=7) responders to BCG. In freshly isolated NK cells*,* high responders had 2.5-fold more HLA-DR-expressing NK cells (*p*=0.03, Fig. 5D). Thus, the size of the circulating population of HLA-DR-expressing NK cells can be associated with IFN-γ secretion by NK cells in PBMCs stimulated with BCG.

To test the role of HLA-DR-expressing NK cells directly, we repeated the BCG stimulations using PBMCs enriched with autologous HLA-DR-expressing NK cells, obtained by magnetic separation of the HLA-DR^+^ fraction of NK cells previously isolated from PBMCs. Importantly, a relatively modest enrichment of HLA-DR-expressing NK cells (median 44% increase, Fig. 5E and F) was sufficient to enhance the NK cell IFN-γ response by over three-fold (median 3.3-fold increase, *p*=0.008, Fig. 5G and H). Thus, although the HLA-DR-expressing NK cells are a small population in ex vivo PBMCs, they can significantly influence immune responses to BCG.

## Discussion

The expression of HLA-DR on NK cells has previously been used as a marker of cellular activation 10–17. However, the expression of HLA-DR was only observed on a proportion of NK cells and took 3 days or more, which is not consistent with HLA-DR being a simple marker of NK cell activation. This is in contrast with other activation markers, such as CD69, which is expressed on the vast majority of activated cells within 18 h of IL-2 stimulation 23. By following proliferation of human NK cells (via dilution of CFSE), we demonstrate here that HLA-DR is expressed on cells that proliferate in response to IL-2 or IL-15. In addition, NK cells derived by clonal expansion of a single seed cell were relatively homogeneous in their expression of HLA-DR. These data suggest that expression of HLA-DR after culture of NK cells in IL-2 is due to the expansion of a distinct subset of HLA-DR-expressing NK cells. This model was confirmed by our finding that NK cells sorted for HLA-DR expression proliferate far more readily than those lacking HLA-DR expression ex vivo*.*

There is considerable precedence for plasticity in NK cell subsets. For example, IL-21 and IL-2 together can induce upregulation of CD56 on CD56^dim^ NK cells 9. In addition, after 7 days co-incubation with LPS-matured DCs, most NK cells have been shown to express HLA-DR 19. Our data do not exclude the possibility of de novo expression of HLA-DR on NK cells, particularly following other stimuli. Nevertheless, an important implication is that the large volume of human NK cell research that uses peripheral blood NK cells expanded in IL-2 involves preferential expansion of a small subset of NK cells. Functional analyses of such cells will be skewed towards the responses of the HLA-DR-expressing NK cells rather than the bulk of circulating NK cells.

These data also suggest that the accumulation of HLA-DR-expressing NK cells at sites of inflammation 10, 19 may reflect CXCR3-mediated recruitment or in situ expansion of the HLA-DR-expressing NK cells, rather than NK activation per se. Moreover, the human HLA-DR-expressing NK cell subset identified here could be analogous to the population of murine cells initially designated IFN-producing killer DCs 37, 38, which are now more properly thought of as a subset of activated NK cells 39, 40.

An important goal is to address the functional importance of HLA-DR-expressing NK cells. It has been shown previously that NK cells expressing HLA-DR can present antigen to trigger T-cell proliferation and IL-2 production 18, 19. The accumulation of HLA-DR-expressing NK cells at sites of inflammation 10, 19 and correlations between their numbers and the magnitude of immunopathology (for example in IgA nephropathy 20) suggests that they may contribute to inflammation and immunopathology, either by antigen presentation or an alternative mechanism. In support of this, we have observed that the HLA-DR-expressing NK cells exhibit a significantly stronger cytolytic activity, compared with other NK cells. This corresponds with the described “presentation after killing” mechanism of antigen acquisition via internalisation of engaged NK cell activating receptors and subsequent trafficking to class II MHC protein-loading compartments 41.

To fulfil a role as APCs, HLA-DR-expressing NK cells would need to encounter antigen-specific T cells. For conventional APCs this most frequently occurs in secondary lymphoid organs, such as lymph nodes. Importantly, HLA-DR^+^ NK cells have been reported to account for ∼50% of NK cells in lymph nodes 22 consistent with our observation here of high levels of expression of the lymph node-homing receptor CXCR3 on them. Thus, as in mice 8, 27, CXCR3 may play an important role in recruiting HLA-DR-expressing NK cells to secondary lymphoid organs.

One important area of contemporary immunology is to understand and parameterise heterogeneity amongst human immune systems and responses 42. One recent example is the considerable heterogeneity amongst naïve donors in NK cell IFN-γ secretion in response to malaria-infected erythrocytes 35. This NK cell secretion of IFN-γ can be inhibited by blocking MHC class II proteins or IL-2, and by depleting antigen-specific CD4^+^ T cells, thus positioning the IFN-γ-secreting NK cells as important regulators during an antigen-specific recall response 32–34. Here, we found a similar dependency on MHC class II proteins, CD4^+^ T cells and IL-2, consistent with the NK cell secretion of IFN-γ in response to BCG stimulation acting as an important regulator mechanism during the adaptive immune response. Moreover, by specifically enlarging the HLA-DR-expressing compartment, NK cell secretion of IFN-γ in response to BCG could be enhanced. It can be speculated that this is due to antigen presentation by HLA-DR-expressing NK cells, which could perhaps be mediated by uptake via the NK cell activating receptor NKp44, which can bind components of the mycobacterial cell wall 43, but this remains to be tested specifically.

It is intriguing that there was significant heterogeneity amongst donors in the NK cell secretion of IFN-γ in response to BCG. This heterogeneity was associated with the size of the circulating HLA-DR-expressing NK cell population, which suggests that heterogeneity in human immune responses to BCG, at least in vitro, can be linked to the size of the HLA-DR-expressing NK cell compartment in peripheral blood. This gives a novel suggestion to why individuals consistently respond differently to BCG and other pathogens, and could have important implications in understanding differences between donors in the efficacy of the BCG vaccine. Taken together, these data suggest a specific and important immunological function for the subset of human NK cells that express HLA-DR.

## Materials and methods

### Cells

Healthy adult donors gave fully informed consent for their blood to be used in this study, which was approved by the London School of Hygiene and Tropical Medicine and by the Riverside NHS research ethics committees as appropriate. Venous blood was collected into sodium heparin (10 IU/mL blood; CP Pharmaceuticals), and PBMCs were isolated by Histopaque 1077 (Sigma-Aldrich, St. Louis, MO) density gradient centrifugation as described previously 44. NK cells were isolated from PBMCs by negative selection using magnetic activated cell sorting (LS columns, Miltenyi Biotech).

Where specified, NK cells were depleted from PBMCs using CD56 microbeads, or HLA-DR^+^ NK cells were separated from NK cells using HLA-DR microbeads, followed by magnetic separation (Miltenyi Biotech). Isolations and depletions were confirmed by flow cytometry for expression of CD3, CD56 and HLA-DR. NK cells were cultured in X-vivo-10 media (Lonza) supplemented with 10% heat-inactivated FCS with recombinant (r)IL-2 (200 U/mL, Roche), rIL-12 or rIL-15 (both 10 ng/mL, R&D systems). Freshly isolated human NK cells were sorted into CD56^+^/HLA-DR^+^ and CD56^+^/HLA-DR^−^ populations by flow cytometric cell sorting (BD FACS Aria II). The percentage of HLA-DR-expressing NK cells in a polyclonal population after 6 days culture with IL-2 that derived from the small initial population of HLA-DR-expressing NK cells ex vivo was calculated by factoring the percentage of HLA-DR^+^ NK cells (%DR^+^) in each sorted population (subscript _DR−_ or _DR+_) by the relative population size (rel.pop.), according to the following equation,


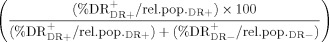


NK cell clones were generated from isolated NK cells as described previously 44. Briefly, freshly isolated NK cells were seeded by limiting dilution in 96-well round-bottom plates and cultured for 4 wk. K562 cells were cultured in RPMI supplemented with 10% heat-inactivated FCS, 1% penicillin/streptomycin and 1% l-glutamine (both Gibco).

### Flow cytometry

Samples were labelled with directly conjugated mouse mAbs in 2% BSA/0.01% sodium azide/PBS for 30 m on ice. The following antibodies were purchased from BD Biosciences: anti-CD3 FITC (clone UCHT1, IgG1), anti-CD3 PE/APC (clone HIT3a, IgG2a), anti-CD56 FITC/PE/APC (clone B159, IgG1), anti-HLA-DR FITC/PerCP (clone L243, IgG2a; confirmed with clone TÜ36, IgG1), anti-IFN-γ APC (clone B27, IgG1). In addition, the following antibodies were used: anti-CD69 APC (Biolegend, clone FN50, IgG1), anti-CXCR3 APC (R&D Systems, clone 49801, IgG1). Samples were acquired on a FACSCalibur (BD Biosciences) and data analysed using FlowJo software (TreeStar, OR). Propidium iodide (Invitrogen) was used for live/dead cell discrimination. Freshly isolated human NK cells were labelled with 1 μM CFSE for 15 m at 37°C in RPMI, then washed three times before stimulation in culture media. CFSE dilution in proliferating cells was analysed by flow cytometry.

### Cell stimulations

Purified PBMCs were plated at 2×10^6^ cells/mL in 96-well round-bottom plates and stimulated with BCG for 24 h at a MOI (BCG:PBMC) of 10:1, the optimal ratio determined by titration (Supporting Information Fig. S7). Samples were analysed for expression of CD3, CD56, HLA-DR and IFN-γ by flow cytometry.

### Functional assays

To assay for NK degranulation, NK cells were co-incubated with K562 target cells for 1 min, 1 h or 2 h with PE-conjugated anti-CD107a mAbs included in the media (clone H4A3, IgG1, BD Biosciences). Samples were harvested, stained for surface markers, fixed with 2% PFA/PBS and analysed by flow cytometry. To determine secretion of IFN-γ, NK cells were incubated with K562 target cells or PMA (50 ng/mL)/ionomycin (1 μg/mL, both Sigma Aldrich) for 5 h. Aliquots of 10 μg/mL Brefeldin A (Sigma Aldrich) were added to the wells 4 h before samples were harvested. Samples were stained for surface markers, then fixed and permeabilised (CytoFix and PermWash buffer, BD Biosciences), before staining for intracellular IFN-γ and analysed by flow cytometry. Chemotaxis assays were performed as previously described 45, using ChemoTX plates with a 5 μm pore size, (Neuroprobe, Gaithersburg, MD) and the chemokine I-TAC (PeproTech, EC). For NK cell migration, enumeration was carried out using a haemocytometer, with individual results expressed as a percentage of the total cells applied to the filter. The phenotype of the responding NK cells was analysed by flow cytometry. Enrichment of the HLA-DR^+^ NK cells was calculated by subtracting the percentage of HLA-DR^+^ NK cells that migrated in the buffer-only sample, from that at each chemokine concentration, and expressed as a percentage of the buffer-only control for comparison. CXCR3 internalisation was measured by analysing surface expression of CXCR3 by flow cytometry on the migrated NK cells. The intensity of expression (calculated as geomean of fluorescence) was compared with that in the buffer-only control sample.

## Abbreviation

BCG: Mycobacterium bovis BCG
PFA: paraformaldehyde

## Supplementary material

Detailed facts of importance to specialist readers are published as ”Supporting Information”. Such documents are peer-reviewed, but not copy-edited or typeset. They are made available as submitted by the authors.
