# Supplementary material for: A distinct subset of human NK cells expressing HLA-DR expand in response to IL-2 and can aid immune responses to BCG
Source: Eur J Immunol. 2011 Apr 14;41(7):1924–33. doi: 10.1002/eji.201041180 (PMC3549558; doi:10.1002/eji.201041180)
Supplement: Supplementary file 1 [file eji0041-1924-SD1.pdf]

European Journal of Immunology

## Supporting Information

for

DOI 10.1002/eji.201041180

# A distinct subset of human NK cells expressing HLA-DR expand in response to IL-2 and can aid immune responses to BCG

J. Henry Evans, Amir Horowitz, Maryam Mehrabi, Emma L. Wise, James E. Pease, Eleanor M. Riley and Daniel M. Davis

© 2011 WILEY-VCH Verlag GmbH & Co. KGaA, Weinheim 

# Supplemental Figure S1

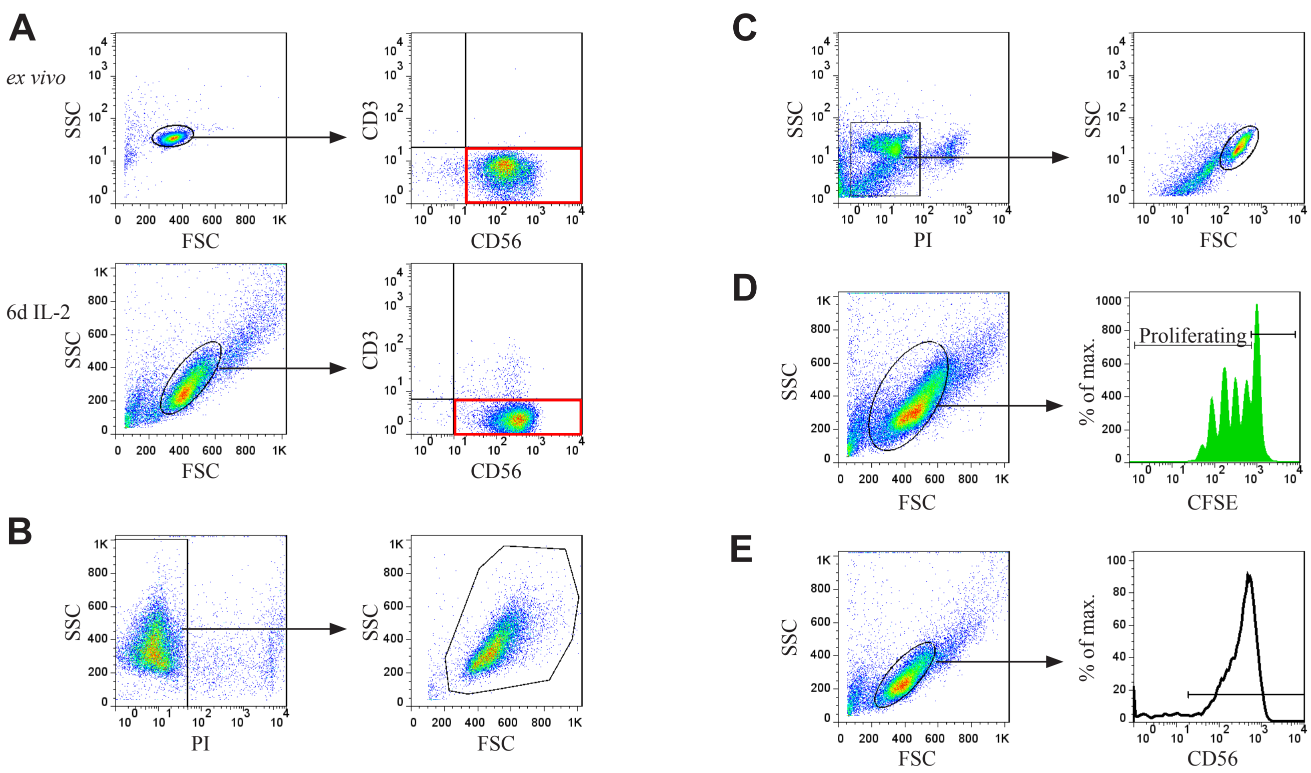


**Supplemental figure S1. Gating strategies for flow cytometric data in Figure 1.**

(A) Gating used to identify NK cells for analysis of expression of HLA-DR in Fig. 1A. Freshly isolated human NK cells were analysed by flow cytometry *ex vivo* (top) and after 6d culture with rhIL-2 (bottom). NK cells were gated in the FSC/SSC plot (left) and by expression of CD56 but not CD3 (right), as indicated by red frame. (B) Gating used to identify NK cells for analysis of CFSE concentration and expression of HLA-DR and CD69 after 6d culture with IL-2 in Fig. 1B. NK cells were gated as $PI^-$ (left) and in the FSC/SSC (right), as indicated. (C) Gating used to identify NK cells for analysis of CFSE concentration and expression of HLA-DR and CD69 after 2d culture with IL-2 in Fig. 1C. NK cells were gated as $PI^-$ (left) and in the FSC/SSC (right), as indicated. (D) Gating used to identify NK cells for analysis of CFSE concentration after 6d culture with IL-15 or IL-12 in Fig. 1E-F. NK cells were gated in the FSC/SSC (left), as indicated, and proliferating NK cells were defined as those with diluted CFSE, marked on the plot (right). (E) Gating used to identify NK cells for analysis of HLA-DR expression after 6d culture with IL-15 or IL-12 in Fig. 1E,G. NK cells were gated in the FSC/SSC (left), and as $CD56^+$ (right), as indicated.

Supplemental Figure S2

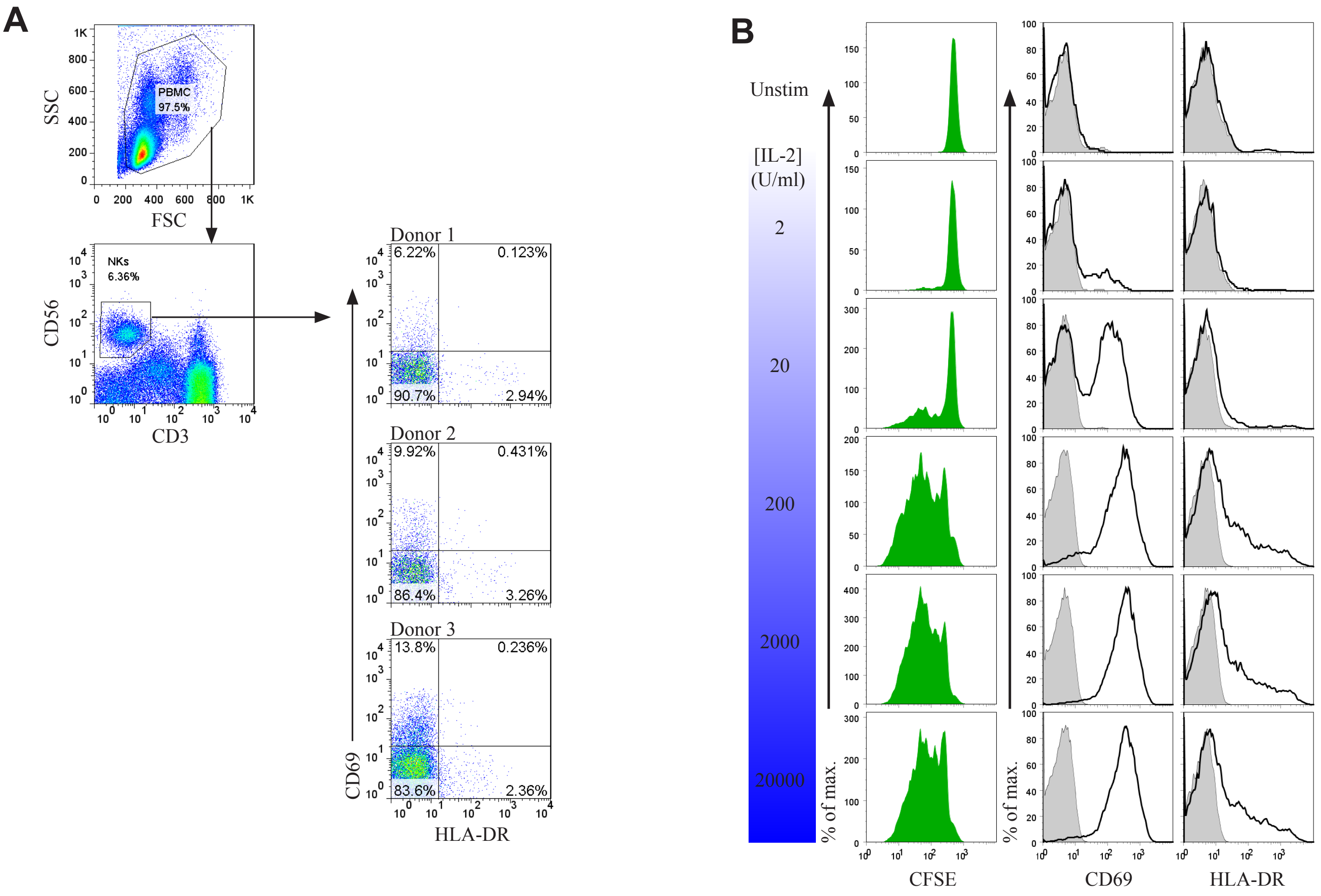


**Supplemental figure S2. Comparison of the activation markers CD69 and HLA-DR.**

(A) Freshly isolated human PBMC were analysed by flow cytometry for expression of CD56, CD3, CD69 and HLA-DR. As indicated, lymphocytes were gated in the FSC/SSC plot, NK cells were gated by CD56$^{+}$/CD3$^{-}$, and the expression of CD69 and HLA-DR were compared. Data displayed for 3 independent donors. (B) Freshly isolated human NK cells were labelled with CFSE and cultured for 6d with rIL-2 at different doses (as indicated) or without rIL-2 (top). The cultured NK cells were harvested and analysed by flow cytometry for CFSE intensity (left), to determine proliferation, and expression of CD69 (middle) and HLA-DR (right). Histograms display isotype control (grey shaded) and specific staining (black). Representative of 2 experiments.

# Supplemental Figure S3

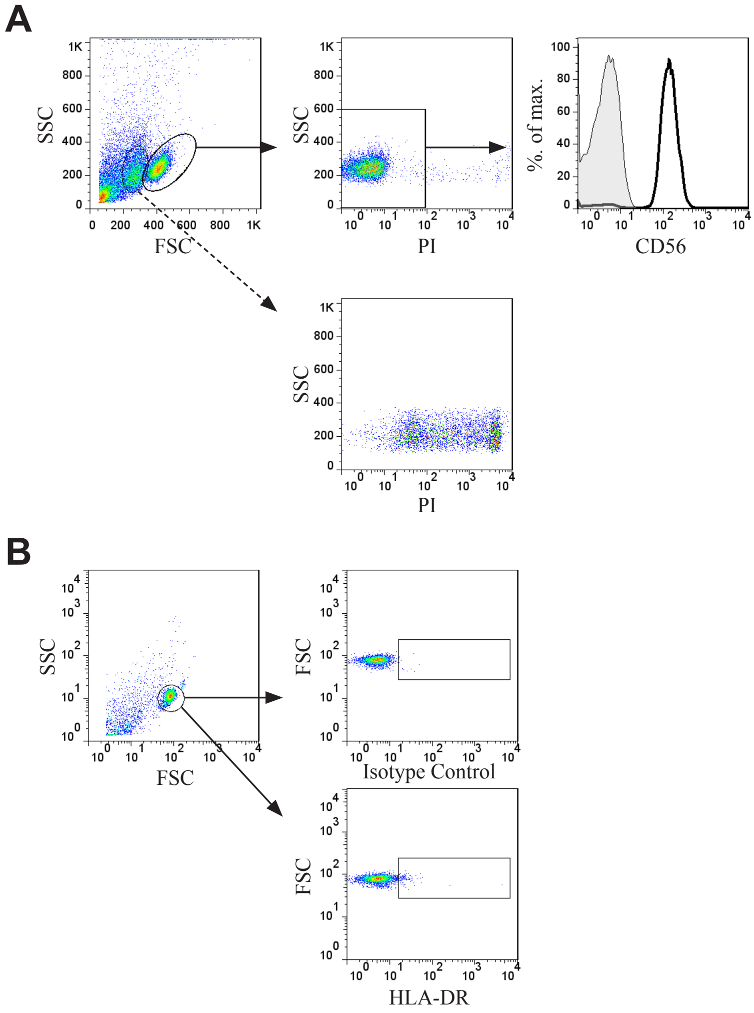


**Supplemental figure S3. Gating strategies for Figure 2, identifying clones and validating cell sorting.**

(A) NK cell clones were prepared from isolated NK cells by limiting dilution and cultured for 4 weeks. Clonal populations were analysed by flow cytometry for expression of CD56 and HLA-DR. Live clonal populations were identified in the FSC/SSC plot (left) and confirmed as live by absence of PI staining (middle top). Expression of CD56 was confirmed on the clones (right). The second distinct population in the FSC/SSC plot was identified as apoptotic/dead cells by PI staining (middle bottom). (B) Expression of HLA-DR on NK cells was tested after cell sorting NK cells for those that lacked expression of HLA-DR. Expression of HLA-DR (bottom) was compared to staining with the isotype control (top). Nunbers indicate percentage of positive staining NK cells.

# Supplemental Figure S4

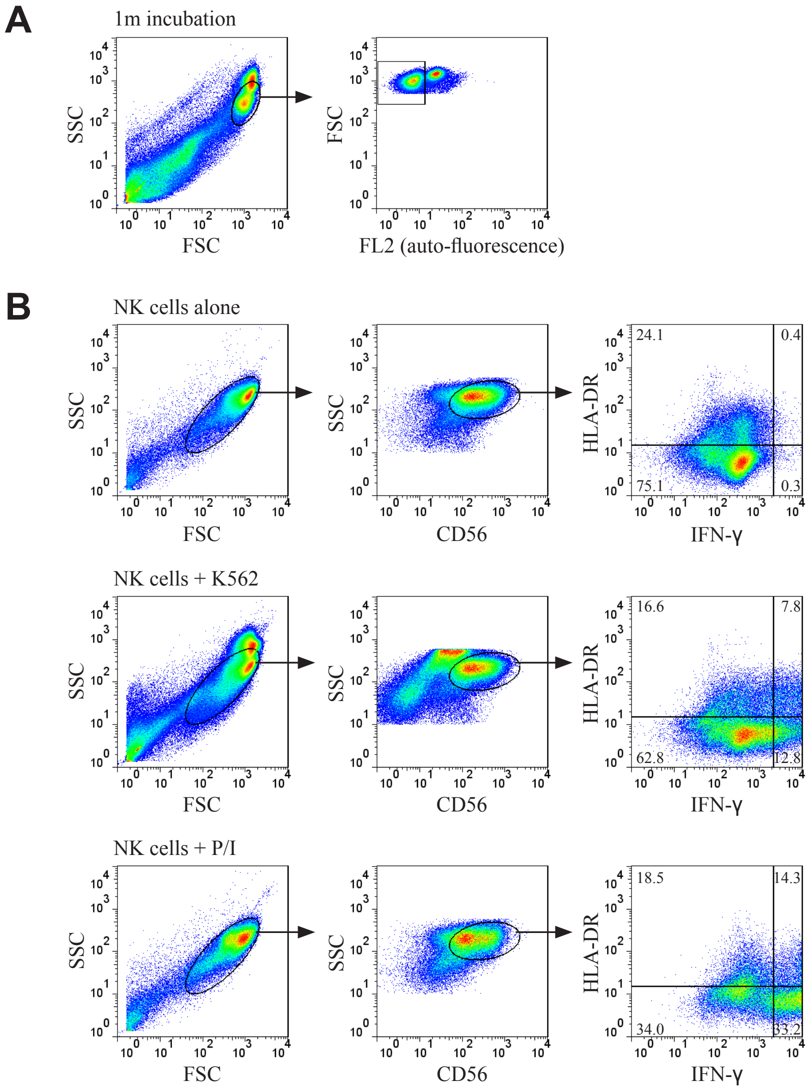


**Supplemental figure S4. Gating strategies for Figure 3, functional assays.**

(A) NK cells were cultured for 6d with IL-2 and used in a degranulation assay with K562 target cells, as shown in Fig. 3A-B. NK cells were distinguished from K562 target cells in the FSC/SSC plot (left) and by the auto-fluorescence of K562 cells in FL2 on a FACScalibur (right). (B) NK cells were cultured for 6d with IL-2 and then cultured with K562 target cells and analysed by flow cytometry for intracellular IFN-γ, as shown in Fig. 3C. NK cells were distinguished from K562 target cells in the FSC/SSC plot (left) and by expression of CD56 (middle). Intracellular IFN-γ was compared on HLA-DR positive and negative NK cells (right).

# Supplemental Figure S5

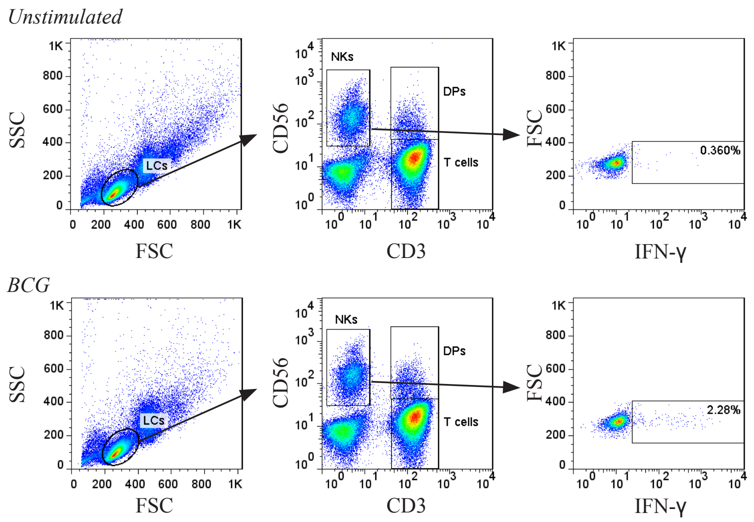


**Supplemental figure S5. Gating strategies for Figure 5, intracellular IFN-γ detection in NK cells in PBMC to stimulation with BCG.**

To quantify the proportion of NK cells in human PBMC than produce IFN-γ in response to stimulation with BCG freshly isolated human PBMC were left unstimulated (top), or stimulated with BCG (bottom) for 24h, and then analysed by flow cytometry for intracellular IFN-γ. NK cells were identified by gating in the FSC/SSC (left) and for $CD56^{+}/CD3^{-}$ in the lymphocyte population (middle), as indicated by arrows.

# Supplemental Figure S6

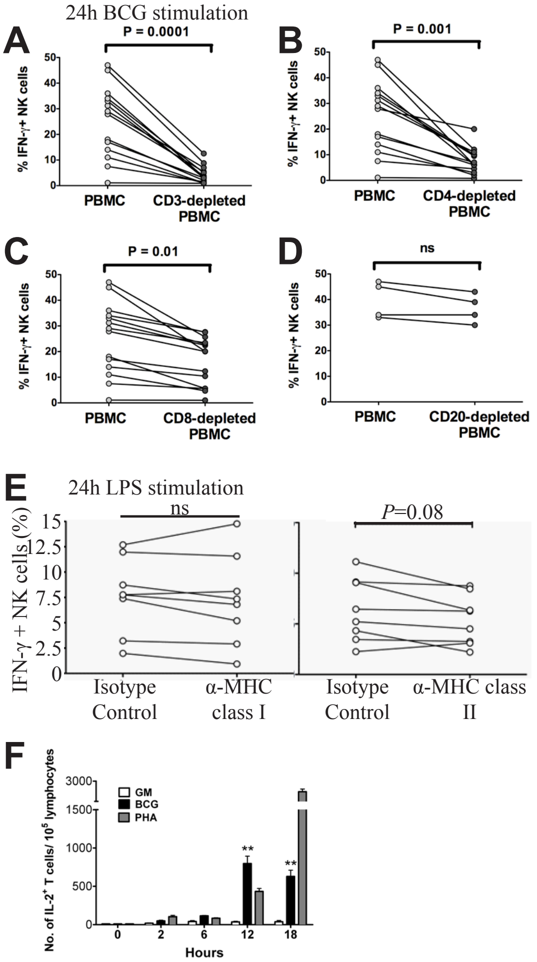


**Supplemental figure S6. NK cell responses to *M. bovis* BCG are dependent upon CD4$^{+}$ T cell help.**

Intact PBMC or PBMC depleted of various T cell subsets were incubated for 24h with (A-D) BCG or (E) LPS, and the percentages of NK cells staining positive for IFN-γ were measured by flow cytometry.  PBMC were depleted of (A) all CD3$^{+}$ cells; (B) CD4$^{+}$ T cells; (C) CD8$^{+}$ T cells and (D) CD20$^{+}$ B cells to demonstrate the effects of depleting an irrelevant cell subset. PBMC were also incubated with  (E) LPS in the presence of blocking antibodies to MHC class I or MHC class II molecules or their respective isotype control antibodies. P values are for 2-tailed paired Wilcoxon tests, 95% CI, for 15 donors (BCG) or 8 donors (LPS). (F) PBMC were incubated with GM (white bars), BCG (black bars) or PHA (grey bars) for up to 18h and the numbers of T cells staining positive for intracellular IL-2 were measured by flow cytometry. P values are for 2-tailed paired Wilcoxon test, 95% CI, for 5 donors.

# Supplemental Figure S7

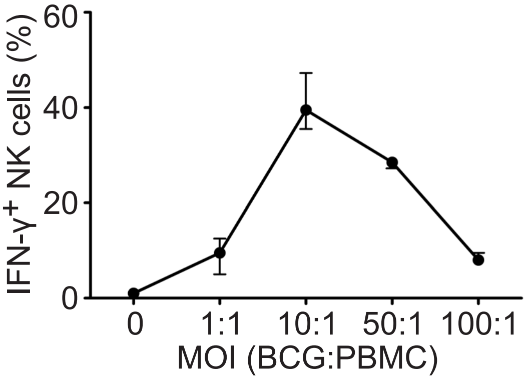


**Supplemental figure S7. Titrating M. bovis BCG input dose to define optimal multiplicity of infection for NK cell activation.**

The proportion of all NK cells expressing intracellular IFN-γ after 24h culture of PBMC with BCG was measured at varying multiplicities of infection (MOI, BCG:PBMC) in 5 donors. Graph shows medians and ranges.
